# An Address to the American People

**Published:** 1899-10

**Authors:** M. L. Lockwood

**Affiliations:** President, American Anti-Trust League


### AN ADDRESS TO THE AMERICAN PEOPLE.

Office of M. L. Lockwood, National President, The American Anti-Trust League, Zelienople, Pa., July 17th, 1899.

The object of the American Anti-Trust League is to drive from public places the subservient tools of the trusts, monopolies, combines, and corporations, and to establish the equal rights of American citizens in the commerce and industries of the country.

To-day in every legislative hall, both state and national, omnipresent stands the lobbyist and corruptionist of this great railroad, monopoly, trust combination, which has formed an alliance offensive and defensive, by which they expect to control legislation and to plunder the producers and consumers of America.

And, what is still worse, there also stand the representatives, elected by the people, who have become debauched and who are the secret servants and instruments of this great corporate power and are ever ready to do their bidding, while proclaiming their devotion to the rights of the people. We find them everywhere—in the Legislature, in Congress, in the Senate, and on the Bench. The political life of this kind of representatives is made easy. Their renominations are arranged for them by the political bosses, who are but the servants of this power. Campaign funds are furnished. That part of the public press that can be managed is used to eulogize and lionize these subservient tools. Their elections are managed for them.

There also stands the representative elected by the people, who is true to the principles of manhood and is governed by the promptings of public welfare. He stands a bulwark against the legislative schemes by which this great railroad, monopoly, trust combination expect to plunder the people. He is obnoxious to this dominating and controlling power, and their emissaries are sent into his district to undermine and destroy him politically. Some popular man is encouraged to become a condidate for nomination to his place, and the power and influence and money of this corporate conspiracy are put behind this candidate, and the true servant of the people finds himself defeated for renomination, and he is retired from public life—retired because he was a true representative of the people and dared to defy this corporate monster. This process has been worked so silently and secretly that the people have not recognized the handiwork by which a majority in their legislative bodies have been controlled.

In many of our great cities the street railway traction companies have created a political despotism. The man who is ambitious politically, before he can hope to be nominated to any position of public trust must first kneel at the throne. Yes, and in many, many cases before a laborer can hope to earn bread for his family he has first to show that he wears the brand and collar of their ward boss. They have created a despotism so damnable that man must become a serf to this corrupt corporate power before he can obtain an opportunity to work. I have talked with these men who mourn the loss of their liberty as American freemen. This great railroad, monopoly, trust, traction combination is "corrupting our public affairs and debauching our public men" and destroying the foundations of the Republic by the corrupt use of money in our political life.

The purpose of the American Anti-Trust League is to arouse the only power on earth that is stronger than the power of money in our public life. That power is the patriotic impulses of the people. The little finger of that power, when awakened is stronger ten thousand times than the influence of all the billions of the trusts and combines of the land. The memories of the many sacrifices of the fathers call us to action. If these trust combinations are allowed to go on they can plunder each of us into poverty. No man knows how soon the fear of hunger for his wife and family will make him a coward. It behooves us to strike while the fire of liberty yet burns.

The American Anti-Trust League is non-partisan. We call all American freemen to council. If a Democrat, or a Populist, or a Republican, public man has shown himself to be a subservient tool of this great corporate power, then all the united power of all the men of the American Anti-Trust League will be used to crush him and drive him from public life. We will adopt the tactics of our enemy until we have created a legislative, judicial, and executive power in sympathy with the public welfare. And we call upon every American citizen who loves his country and the great principles of popular government better than he does his party, to join us in the work of re-establishing the equal rights of American citizenship.

M. L. Lockwood,

*President, American Anti-Trust League.*

"*Salus Populi est Suprema Lex.*"

---

Whortleberry in Eczema.—Daxenberger, in a German journal, speaks very favorably of the use of the extract of whortleberry in various forms of eczema, especially of children, also in the myotic forms in smaller ulcerations of the skin and mucous membranes. A syrup of the extract is applied with a brush after the surface has been cleansed and dried; over this any simple dusting powder may be applied. Drying speedily takes place and no other dressing is required. The cure is often effected in two or three days.—*Medical Times, Sept., 1899.*

